# Transcriptomic Analysis of the Regulation of Lipid Fraction Migration and Fatty Acid Biosynthesis in *Schizochytrium* sp.

**DOI:** 10.1038/s41598-017-03382-9

**Published:** 2017-06-15

**Authors:** Lujing Ren, Xuechao Hu, Xiaoyan Zhao, Shenglan Chen, Yi Wu, Dan Li, Yadong Yu, Lingjun Geng, Xiaojun Ji, He Huang

**Affiliations:** 1 Jiangsu National Synergetic Innovation Center for Advanced Materials, Nanjing Tech University, No. 30 South Puzhu Road, Nanjing, 211816 People’s Republic of China; 2 College of Biotechnology and Pharmaceutical Engineering, Nanjing Tech University, No. 30 South Puzhu Road, Nanjing, 211816 People’s Republic of China; 3 School of Pharmaceutical Sciences, Nanjing Tech University, No. 30 South Puzhu Road, Nanjing, 211816 People’s Republic of China; 4 Xiamen Kingdomway Group company, No. 299 West Yangguang Road, Haicang, Xiamen 361022 China

## Abstract

*Schizochytrium* sp. is the main source of docosahexaenoic acid-rich oil, which is widely used in food additive and pharmaceutical industry. In this study, using RNA-seq, comparative transcriptomic analyses were performed at four stages of DHA fermentation by *Schizochytrium* sp to get potential genes related to cell transition from cell growth to lipid accumulation and then to lipid turnover. 1406, 385, 1384 differently expressed genes were identified by comparisons in pairs of S2 vs S1, S3 vs S2 and S4 vs S3. Functional analysis revealed that binding and single-organism process might be involve in the cell transition from cell growth to lipid accumulation while oxidation-reduction process played an important role in the transition from lipid accumulation to lipid turnover. pfaC in the PKS pathway showed higher sensitivity to the environmental change, which might be the key regulator for enhancing PUFA biosynthesis in the future. Some other genes in signal transduction and cell transport were revealed to be related to lipid turnover, which would enrich the current knowledge regarding lipid metabolism and help to enhance the DHA production and enrich different lipid fractions by *Schizochytrium* in the future.

## Introduction

Microbial lipid, also called single cell oil (SCO), is a new kind of lipid resource with wide prospect and gets more and more people’s attention in recent years. It is produced by various oleaginous microorganisms which can accumulate more than 30% lipids in the cell^1^. *Schizochytrium* sp., a heterotrophic marine microalga, has attracted much attention due to its ability to produce more than 50% lipid rich in docosahexaenoic acid (DHA, 22:6), an important member of n-3 polyunsaturated fatty acids (PUFAs)^2^. It plays significant roles in enhancing brain cell development and preventing certain cardiovascular diseases^3^. In addition, scientists also found DHA also can reduce blood pressure^4^ and inhibit UVB-induced inflammation in the skin^5^, which expand the application in food additives and nutrition supplement. The traditional commercial source of DHA is fish oil. *Schizochytrium* sp. is noteworthy and considered as a satisfactory alternative to fish oil due to the advantages of fast growth rate and high productivity^6, 7^.

As for the oleaginous microorganisms, higher lipid yield and high DHA percentage in lipid are two premises to realize the industrialization of single cell oil. In recent years, various regulation strategies, including nitrogen limitation^8, 9^, oxygen supply^10–12^, temperature shift^13, 14^, strain adaption^15^ and genetic engineering^16, 17^, were performed to enhance lipid accumulation efficiency in *Schizochytrium* sp. In addition, single cell oil also contains neutral lipid (NLs), phospholipids (PLs), glycolipids (GLs) and unsaponifiable matters (UMs) and its synthesis involves multiple metabolic pathways and enzymes^18^. Besides, people also found the shift of neutral lipid to polar lipid and the increase of unsaponifiable matters happened in lipid turnover stage^19, 20^, which was closely related to the lipid quality. Therefore, lipid accumulation and lipid turnover are two important but complex bioprocesses.

To uncover the mystery of lipid accumulation and migration, many efforts were done in recent years. The whole genome of *Schizochytrium* was sequenced^21–23^ and the genome-scale metabolic model was reconstructed and analyzed to elucidate the mechanism of DHA biosynthesis^24^. A number of enzymes involved in the formation, storage and degradation of neutral lipids have been identified at the molecular level^25, 26^. But the above researches are limited in a certain enzyme or conducting the statistical analysis of the whole genome, very few researches have been reported on the global analysis of the transcripts to reveal the mechanisms underlying lipid accumulation and migration, not to mentation the control mechanism of polyunsaturated fatty acid synthesis.

RNA sequencing (RNA-Seq) techniques which can provide massive sequence data for analysis of gene expression have proven to be advantageous and economical for transcriptomic studies^27^. Comparative global transcriptome analysis can deduce a complete view of differentially expressed genes which might closely related to the difference of cultivation time or conditions and can be used to clarify the functions of the corresponding metabolic pathways. Large number of transcriptome studies in many oleaginous microorganisms, such as *Coccomyxa subellipsoidea* ^28^ *Mortierella alpine* ^29^, *Chlorella protothecoides* ^30^ *et al*., have been undertaken, which have proved comparative transcriptome analysis is a powerful approach for discovering the molecular basis underlying specific biological events.

In this study, in order to uncover the regulation mechanism of lipid migration and fatty acid synthesis, we used Illumina’s sequencing technology to examine the transcriptome changes at four stages during DHA fermentation in *Schizochytrium* sp. Furthermore by systematic integrating differentially expressed genes (DEGs) and GO enrichment analyses, several biological processes and pathways were identified to be related to the transition from cell growth to lipid accumulation and from lipid accumulation to lipid turnover. The data generated from this study, will serve as a blueprint of gene expression profile in oleaginous microorganism which will also help to unravel the network of differentially expressed genes related to lipid accumulation, turnover and polyunsaturated fatty acid synthesis.

## Results

### Lipid accumulation and turnover of *Schizochytrium* sp

**Figure 1:**
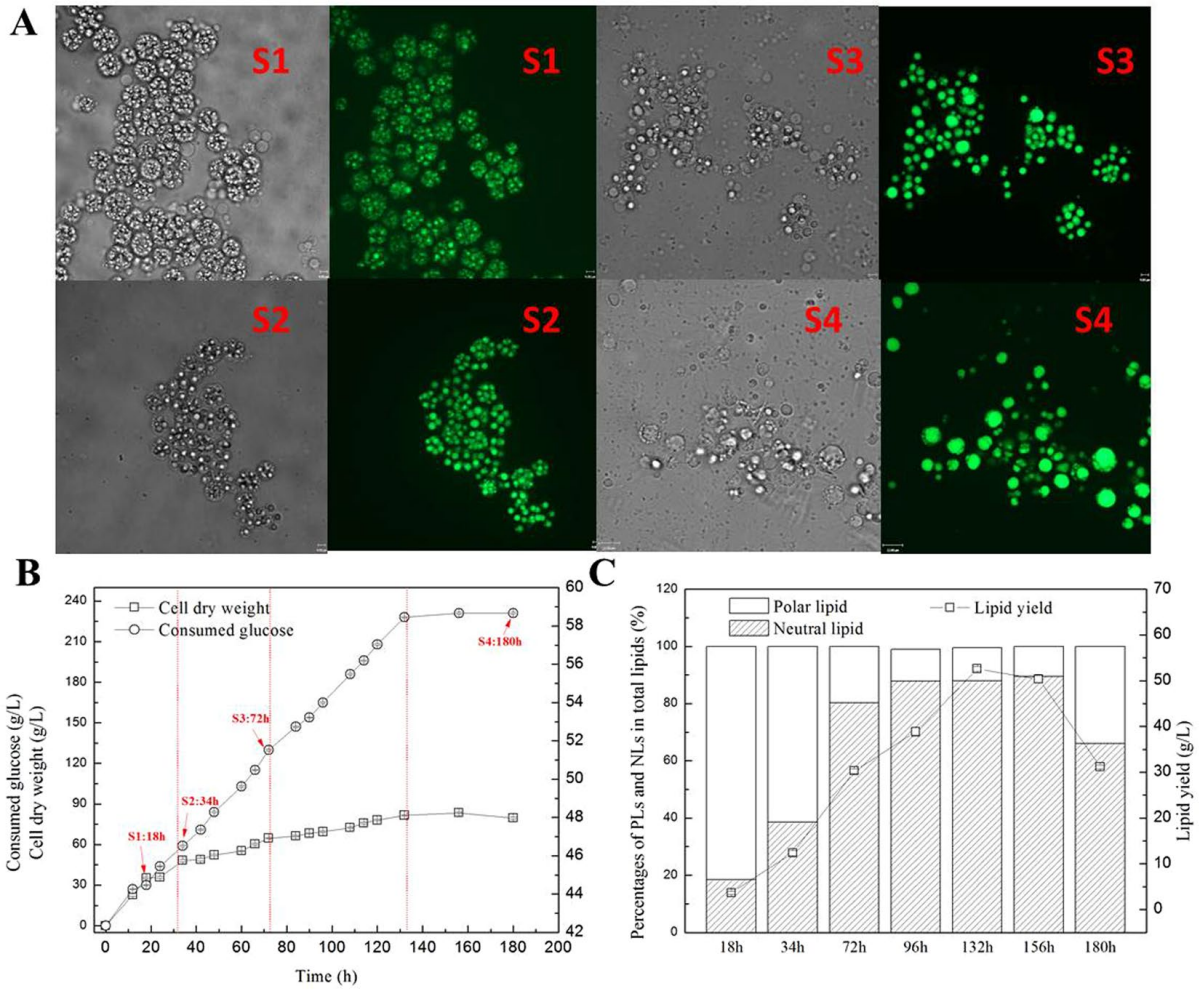
Fermentation characteristic of *Schizochytrium* sp. HX-308 during lipid accumulation and turnover. (**A**) Cell images of *Schizochytrium* sp. HX-308 at different cultivation times, (**B**) Cell growth and glucose consumption, including four stage, cell growth stage (S1), lipid early accumulation stage (S2), rapid lipid accumulation stage (S3), lipid turnover stage (S4); (**C**) lipid yield and lipid fractions at different stages. Each datum is the mean value of three identical samples.

*Schizochytrium* sp. was cultured in a 5L fermenter. Cell growth, lipid accumulation and turnover were monitored by the measurement of cell dry weight, lipid yield, DHA percentage in total fatty acids. In addition, different lipid fractions in total lipids were also analyzed to investigate the compositional shift of lipid fractions. Growth of *Schizochytrium* sp. was divided into four phases. As shown in Fig. 1A, cell division was intense at 18 h and only a little lipid was accumulated in the cell. Dry cell weight grew fast before 32 h because nitrogen source was adequate. After nitrogen exhausted, cells were immersed in a nitrogen limitation environment, which induced cells to accumulate lipid. At this time, cell became single one and lipid droplets can be obviously seen in the cell. After that, lipid yield increased very fast and the size of lipid droplets became even bigger. Lipid yield increased 40.19 g/L during the period from 34 h to 132 h. Glucose exhausted at 136 h and cell entered into the lipid turnover stage, which consumed 21.39 g/L lipid in the following 48 h (Fig. 1B). At this time, cell density decreased a lot but the lipid droplets occupied a large proportion of the cell volume. Besides, the neutral lipid percentage in total lipids at 132 h reached 87.90%. At 156 h, 24 hours after cell entered lipid turnover stage, the value increased slightly but the percentage at 180 h decreased sharply to 66.04% (Fig. 1C), indicating a large part of neutral lipids converted into polar lipids. Interestingly, DHA percentage in total fatty acids kept increasing in the lipid turnover stage (Table 1). These pheonomeon is similar with our previous report^19^ and Chang’s results in *Schizochytrium* sp. S31^31^”

**Table 1:** Fatty acid composition of *Schizochytrium* sp.HX-308 during fermentation.

Time	C14:0	C16:0	Squalene	DPA	DHA
18 h	3.81 ± 0.12	19.74 ± 0.56	0.91 ± 0.02	17.39 ± 0.46	53.39 ± 0.64
34 h	3.85 ± 0.13	17.58 ± 0.47^**^	2.46 ± 0.15^**^	20.03 ± 0.57^**^	51.54 ± 0.59^*^
72 h	3.42 ± 0.14^*^	15.32 ± 0.42^**^	2.56 ± 0.21	20.59 ± 0.48	53.86 ± 0.63^**^
180 h	2.69 ± 0.21^**^	13.54 ± 0.39^**^	1.18 ± 0.17^**^	20.69 ± 0.63	56.32 ± 0.61^**^

Each datum is the mean ± S.D. of three independent experiments replicates. The statistical significance between two adjacent times was presented by t-test, *P < 0.05, **P < 0.01.

### Illumina HiSeq mRNA sequencing

Given our interest in the transcriptional changes that may be involved in regulating lipid accumulation and lipid turnover, we carried out RNA-seq analysis at various time points prior to and after nitrogen exhaustion (sample 1: 18 h and sample 2: 34 h); lipid rapid accumulation stage (sample 3: 72 h, with the highest lipid accumulation rate at this time point) and lipid turnover stage (sample 4: 180 h). 118.8 million reads and 44584 transcripts were generated in total from the four libraries. All the original reads have been deposited in the NCBI Sequence Read Archive (http://trace.ncbi.nlm.nih.gov/Traces/sra/, Accession No. PRJNA381136). More than 95% of clean reads could be aligned against the reference genome of *Schizochytrium* sp. HX-308^21^ by *SOAPaligner/SOAP2*. 8937, 8911, 8881 and 8988 genes were identified in the S1, S2, S3 and S4 libraries, respectively (Table S1). 465–521 novel transcripts and 1003–1253 alternative splicing were found in these four samples. We performed a comparative analysis of the genes predicted and annotated in the four stages. 96.5% genes were commonly expressed in the four stages.

To further investigate the gene expression profiles of the 8750 co-existed genes (Table S2), we performed hierarchical clustering of gene expression levels using the euclidean distance method (Fig. S1). Eight gene clusters were identified with distinction expression patterns. The K1 and K7 clusters contain 108 and 308 transcripts respectively. The expression of these genes was up-regulated at S1 and S3 stages but was extraordinary down-regulated at S2 and S4 stages. The genes in K2 were significantly up-regulated at S3 stage compared to other three stages, whereas, the ones in K2 were significantly down-regulated at S3 stage. The changing tendency of K4 cluster was opposed to K2. The K3 cluster include 1360 genes which showed up-regulation in both S2 and S3 stages indicating these genes might be related to lipid accumulation. K5 and K6 containing 2409 and 1249 transcripts respectively were showing opposite patterns. While cluster 6 showed gradual increase from stage 1 to stage 2 and then kept relative constant, cluster 5 showed gradual decrease from S1 to S2 and remain stable, indicating the key genes involving cell proliferation. The 2938 genes in K8 were all up-regulated at S4 stage compared to other three stages, implying they played an important role in lipid turnover.

### Differentially expressed genes (DEGs) during lipid accumulation and turnover

To further investigate molecular difference during lipid accumulation and lipid turnover stages, differences in gene expression at four stages were examined, and DEGs were identified by pairwise comparisons of the four libraries. Venn diagram was constructed to identify commonly and exclusively regulated genes over the whole fermentation process (Fig. S2A). Comparisons of the four stages identified 1406, 385, 1384 DEGs in pairs of S2 vs S1, S3 vs S2 and S4 vs S3. The number of DEGs detected in the pairs of S3 vs S2 was much lower than the numbers of other two pairs, indicating that there was no big difference in the gene expression pattern at the early and late lipid accumulation stage. More genes were down-regulated than up-regulated in the transitions from S1 to S2 and S1 to S3; on the contrary, the transition from S3 to S4 and from S2 to S4 generated more up-regulated genes than down-regulated genes (Fig. S2B). Considering the less amount of difference genes between S2 and S3, we treated these two stages as the lipid accumulation stage, and compared them with S1 and S4 to obtain the key genes. After analysis, 1092 and 1102 genes were obtained as the common DEGs related to the transition from cell growth to lipid accumulation (S1 vs S2, S1 vs S3) and from lipid accumulation to turnover (S2 vs S4, S3 vs S4), respectively (Table S3).

### Functional classification of DEGs during lipid accumulation and turnover

To depict the functional differences between developmental stages in *Schizochytrium* sp., the gene clusters of DEGs were characterized by GO enrichment analysis to explore the relevant biological functions. As shown in Fig. S3 and Table S4, there was no Go enrichment term between S2 and S3 stages, indicating that there was no big difference in the gene functions between the early and late lipid accumulation stages. There was no common enriched term among other four comparisons in the ontology of bioprocess, but the terms of binding, heme binding and motor activity which belongs to the molecular function category were found in most of the comparisons. And surprisingly, 14/45 GO terms were enriched in S1 vs S2, and among them 9 GO enriched terms in cellular component were common with S1 vs S3. Additionally, single-organism process and single-organism cellular process were found in S1 vs S3. In addition, the terms of oxidation-reduction process was both enriched in S2 vs S4 and S3 vs S4, while lipid metabolic process, lipid biosynthetic process and fatty acid biosynthetic process were all found in S3 vs S4. These results were in consistent with the fermentation results and gene expression profiling, which suggested that cells need to conduct many oxidation-reduction reactions to go through the carbon starvation status and convert the neutral lipids to polar lipids.

### Differential gene expression of central carbon metabolism

**Figure 2:**
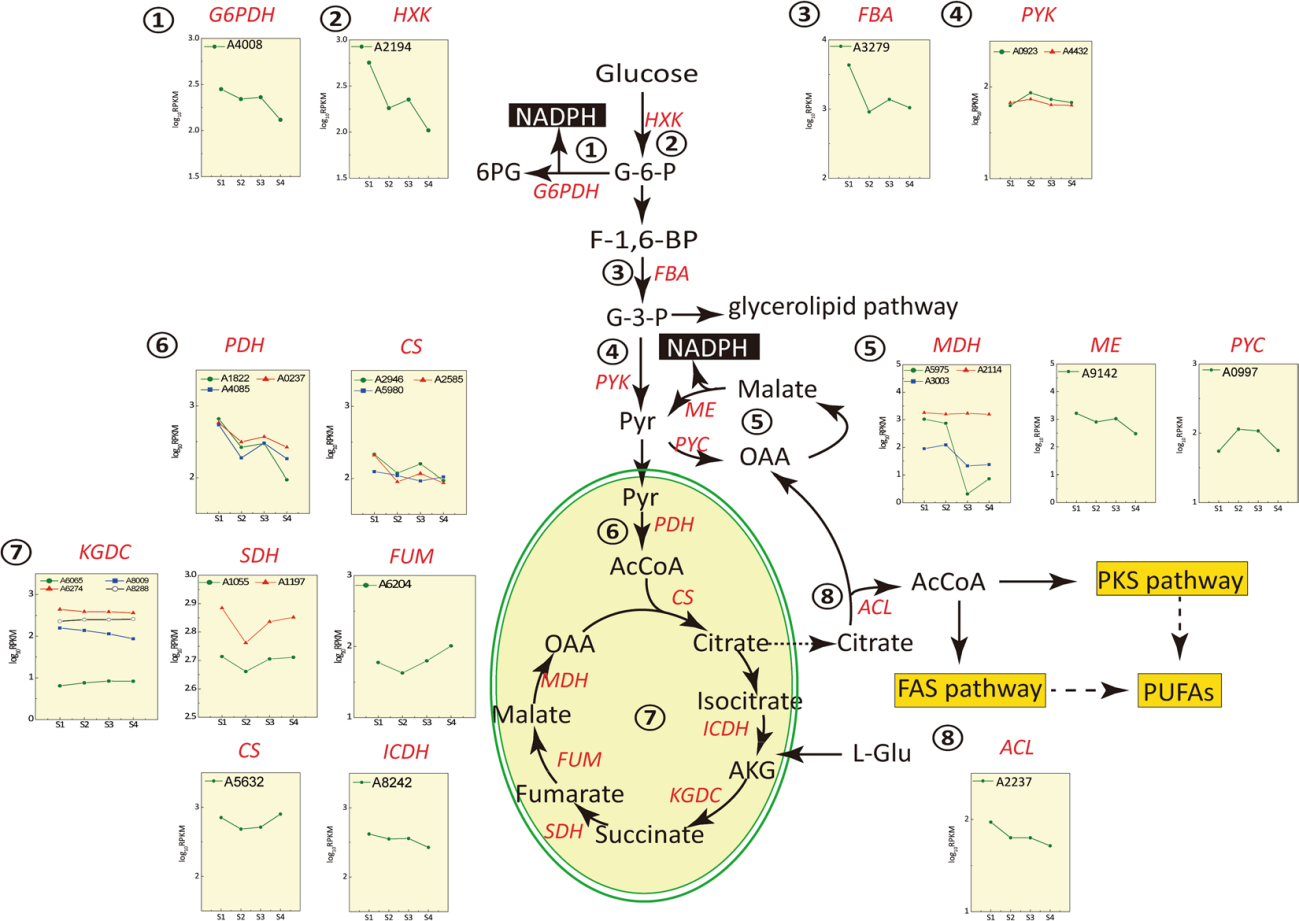
Regulation of NADPH and Acyl-CoA synthesis in *Schizochytrium* sp. HX-308. G6PDH: Glucose-6-phosphate dehydrogenase; HXK: Hexokinase; FBA: Fructose 1,6 bisphosphate aldolase; PYK: Pyruvate kinase; MDH: Malate dehydrogenase; ME: malic enzyme; PYC: Pyruvate carboxylase; PDH: Pyruvate dehydrogenase; KGDC: Ketoglutaricdehydrogenase; SDH: Succinate dehydrogenase; FUM: Fumarate hydratase; CS: Citrate synthase; ICDH: Isocitrate dehydrogenase; ACL: ATP citrate lyase.

Based on the gene annotation information, we tried to identify the gene expression pattern during the transition from cell growth to lipid accumulation and from lipid biosynthesis to lipid turnover. Figure 2 showed the expression changes of genes related to the supply of acetyl-CoA and NADPH, which are two important precursors for lipid accumulation. Malic acid enzyme (ME) and glucose-6-phosphate dehydrogenase (G6PDH) are two important enzymes involving NADPH supply. The expressions of these two genes were down-regulated after entering lipid accumulation stage and then kept at a constant level. We also can see that the expression level of ME was always higher than that of G6PDH, which proved the important roles of ME in NADPH supply. Pyruvate kinase was also found up-regulated after cell entered lipid accumulation stage which produced more pyruvate and ATP for the following reactions. In TCA cycle, citrate might accumulate due to the up-regulation of pyruvate dehydrogenase and citrate synthase and down-regulation of isocitrate dehydrogenase. In addition, the expression of succinate dehydrogenase and fumaric acid hydratase increased while malate dehydrogenase decreased.

### Differential gene expression related to fatty acid biosynthesis

In *Schizochytrium*, saturated fatty acids, such as C14:0 and C16:0 were synthesized by fatty acid synthase, while polyunsaturated fatty acids including DHA and DPA were synthesized by polyketide synthase. As shown in Table 2, the expression changes of FAS, pfaA, pfaB and pfaC showed similar tendency at different stages with lowest value at S4 and highest value at S1. Gene expression of all these four genes decreased 0.28, 0.38, 0.57 and 0.68 times respectively at the shift from S1 to S2. And the log2 ratio (S2/S1) of pfaC was −1.22, which means only this gene has more than two time decrease. Besides that, three kinds of desaturase including Δ-6, Δ-8 and Δ-12 desaturase and one elongase were also detected. Δ-8 desaturase and elongase showed higher expression at the first three stages especially at the stage of S2 while Δ-6 desaturase had higher value at lipid turnover stage. The RPKM value of Δ-12 desaturase kept increasing at the first three stages and reached 3799.5 at S3 then dropped to 663 at S4.

**Table 2:** Differential expression of key genes related to fatty acid biosynthesis.

Gene name	Gene ID	RPKM			
		S1	S2	S3	S4
pfaB	SchizochytriumA1237	490.54	348.60	468.26	51.29
pfaA	SchizochytriumA1238	1587.51	987.24	1153.46	130.93
pfaC	SchizochytriumA5696	1382.43	591.44	1142.01	99.71
FAS	SchizochytriumA3566	531.769	167.98	350.67	171.12
Δ8-desaturase	SchizochytriumA0231	116.66786	161.44131	120.87292	30.923644
Δ8-desaturase	SchizochytriumA4444	0.9719958	0.9027814	0.9896786	2.0838423
Δ8-desaturase	SchizochytriumA6752	48.27933	50.564672	49.727583	48.498036
Δ-12 desaturase	SchizochytriumA6485	2251.7319	2822.5026	3799.4998	663.33732
Δ-6 desaturase	SchizochytriumA1070	9.9669119	1.4547002	4.2525926	13.674547
Δ-6 desaturase	SchizochytriumA2140	9.3371843	4.7950005	2.3138848	10.738789
Elongase	SchizochytriumA4903	62.347467	199.96189	143.94906	79.735939

### Differential gene expression related to glyceride biosynthesis and degradation

**Figure 3:**
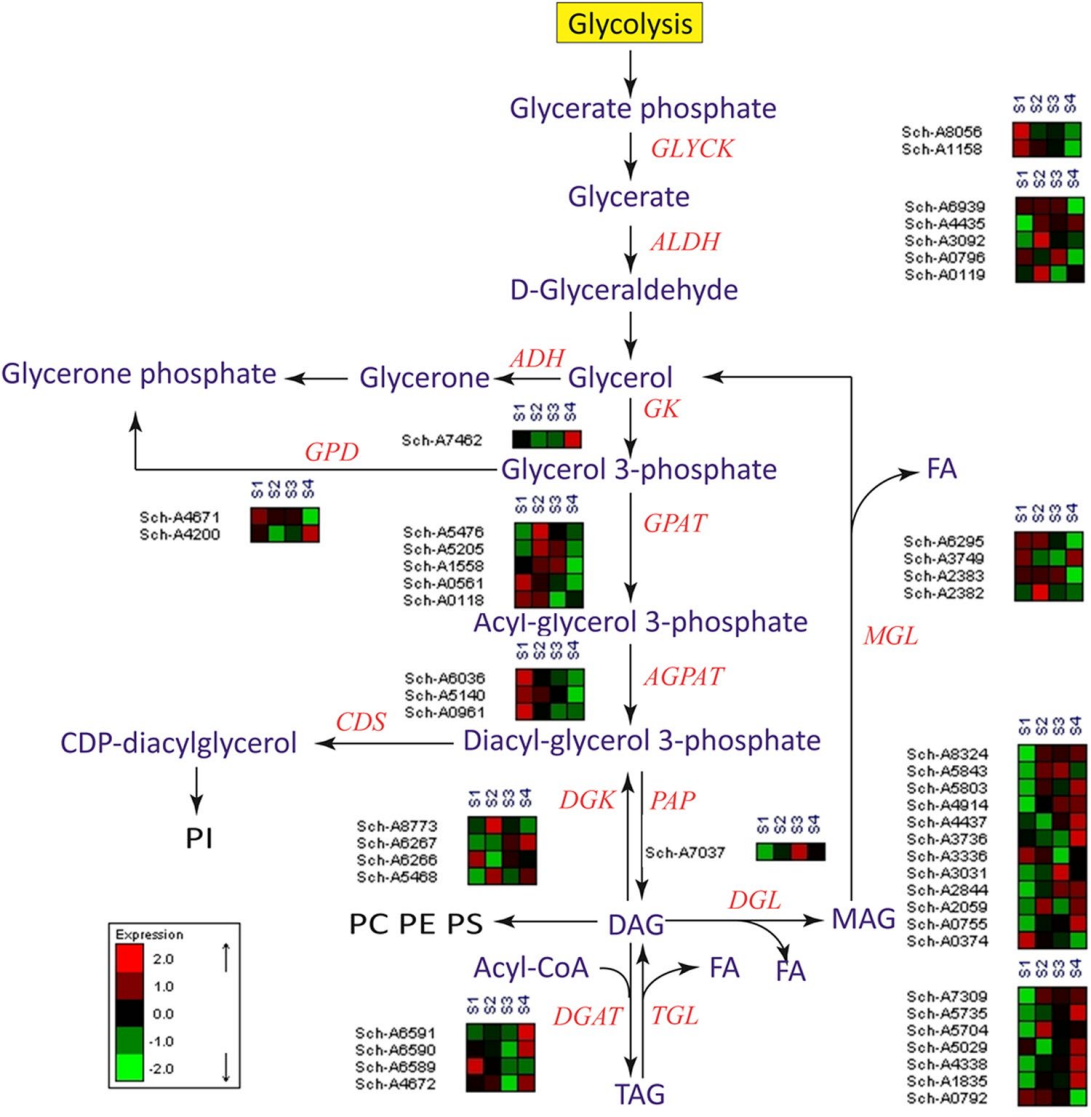
Regulation of key enzymes related to glycerolipid metabolism and glycerophospholipid metabolism in *Schizochytrium* sp. HX-308. GLYCK: Glycerate kinase; ALDH: Aldehyde dehydrogenase; GK: Glycerate kinase; GPAT: Glycerol-3-phosphate-O-acyltransferase; AGPAT: 1-acyl-sn-glycerol-3-phosphate acyltransferase; PAP: Phosphatidate phosphatase; DGAT: Diacylglycerol acyltransferase; DGK: Diacylglycerol kinase; TGL: Triacylglycerol lipase; DGL: Diacylglycerol lipase; MGL: Acylglycerol lipase; GPD: Glyceraldehyde 3-phosphate dehydrogenase.

Genes involved in glyceride biosynthesis and degradation, fatty acid beta-oxidation and glycerophospholipid metabolism pathways are reported to be related to lipid turnover stage. As shown in Fig. 3, the genes in glyceride biosynthesis pathway are GLYCK, ALDH, GK, GPAT, AGPAT, PAP and DGAT. Two homologs of GLYCK, Sch-A8056 and Sch-A1158 (novel gene), had the highest expression at S1 stage and lowest expression at S4 stage. But the gene of GK had opposite expression pattern with the highest expression at S4 stage. GPAT and AGPAT converting glycerol-3-phosphate to diacyl-glycerol-3-phosphate both had the lowest expression at S4 stage. The expression of PAP and DGAT were not the lowest at S4. As for the genes involving lipid degradation, TGL and DGL were both up-regulated at the S4 while the expression of MGL still kept at low level at S4.

**Figure 4:**
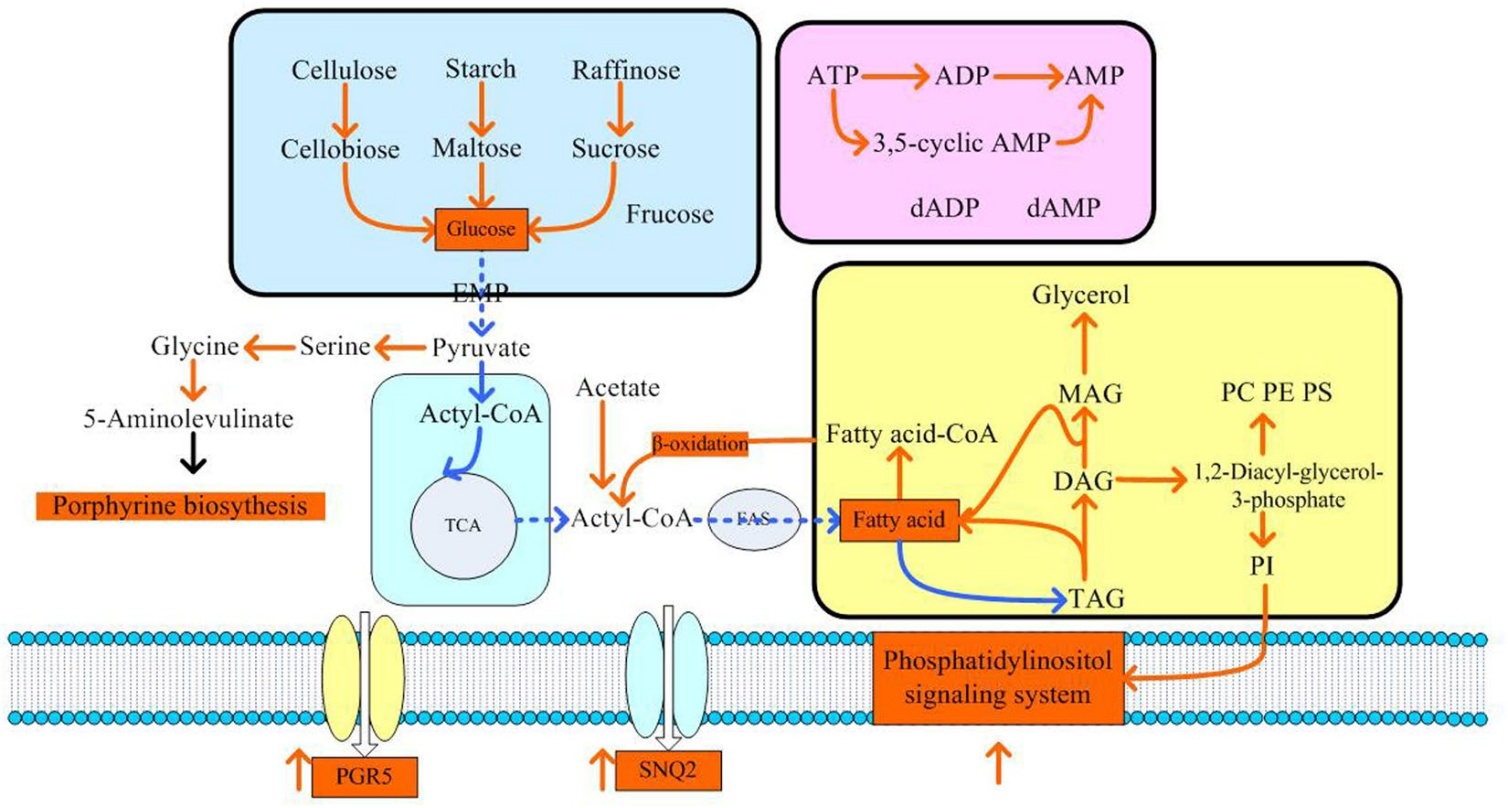
Cell response to the environment of carbon starvation during lipid turnover stage. Yellow: up-regulated; Blue: down-regulated. Abbreviations for genes: MAG: monoacylglycerol; DAG: 1,2-Diacyl-sn-glycerol; TAG: Triacylglycerol; PI: Phosphatidylinositol; PC: phosphatidyl choline; PE: Phosphatidylethanolamine; PS: Phosphatidylserine.

Besides that, some genes concerning glucose regeneration and signal transduction also changed a lot. A series of intracellular reactions were summarized in Fig. 4, α-glucosidase, β-glucosidase and α-galactosidase genes were upregulated to facilitate the glucose formation from cellulose, starch and raffinose to relieve the carbon source shortage. The expression of genes concerning EMP, PPP, TCA, fatty acid synthase and ATP generating pathways were all down-regulated as expected. In addition, some genes related to signal transduction such as the PGR5 and SNQ2 in phosphatidylinositol signaling system, serine deaminase and 5-aminolevulinate synthase catalyzing the reactions from serine to glycine and then to 5-aminolevulinate which will enter to the porphyrine biosynthesis pathway, were also up-regulated in the turnover stage.

### Verification of the gene expression through RT-qPCR

To validate the RNA-seq data, six gene (FAS, PfaA, PfaB, PfaC, acetyl-CoA carboxylase, malic enzyme, 1-acyl-sn-glycerol-3-phosphate acyltransferase, triacylglycerol lipase) associated with lipid and fatty acid biosynthesis were analyzed by qRT-PCR (Fig. S4). The results showed that the gene expression levels detected by RT-qPCR analysis of the selected six genes are consistent with those obtained by transcriptome analysis, thus confirming that the data from the RNA-Seq were reliable.

## Discussion

There is keen interest in the development of technologies that produce microbial lipid rich in polyunsaturated fatty acids. *Schizochytrium* sp. is a potential microalga to realize the industrialization of DHA-rich oil due to its fast growth rate and high DHA percentage in lipid. To further understand and improve the oleaginous phenotype of *Schizochytrium* sp. HX308, we analyzed the transcriptomes of the *Schizochytrium* sp. at different lipid accumulation stages based on the genome database we sequenced before, which provides us a new clue to understand the lipid migration and polyunsaturated fatty acid biosynthesis in microalgae.

Lipid accumulation and degradation are a very hot topic in the lipid research field. Nitrogen limitation are the premise of the lipid accumulation, in our study, most of the genes in central carbon metabolism were down-regulated first after nitrogen depletion (from S1 to S2) and then up-regulated after entering rapid lipid accumulation stage (from S2 to S3), especially some genes related to lipid synthesis, such as HXK, PDH, and FUM. The first decrease at S2 might be related to the poor nutrition and rotational speed decrease and the increase at S3 might ascribe to the gene activation of the lipid accumulation. FUM could synthesis malate which could export to the cytosol for the production of pyruvate by a NADPH-malic enzyme (ME). In Li *et al*.’s transcriptomes study about an oleaginous microalga, *Nannochloropsis oceanica* ^32^, more than 4 fold transcript increase of the fumarate hydratase genes under N-depleted (N−) conditions was found compared with the value under nitrogen-replete (N+) condition, which also proved the importance of this gene. Besides, they also found PDH which converting pyruvate to acetyl-CoA also has the similar tendency resulting in increased precursor for fatty acid synthesis.

About the PUFA synthesis, most people think polyunsaturated fatty acids in *Schizochytrium* sp. were synthesized by PKS pathway, and the standard desaturation-elongation pathway also exit but is incomplete in this strain^33^. Lippmeier *et al*.^34^ found D5, D6 and D9 elongase activities in *Schizochytrium* sp. ATCC20888 but absence of D12 desaturation activity. Minh Hien *et al*.^35^ identified only two unigenes encoding elongase protein and one unigene encoding D6 desaturase. The missing of some specific enzymes might be the reason of the incomplete desaturation-elongation pathway. In our study, we detected one elongase and three kinds of desaturases, Δ-6, Δ-8 and Δ-12, expressed in *Schizochytrium*. Different from Lippmeier’s results, the expression level of Δ-12 desaturase which catalyzes C18:0 to C18:1 was quiet high. Maybe it is the reason why we can detect the C18:1 in our lipid^36^. Similar to Meyer, we also can’t find Δ-4 desaturase, indicating *Schizochytrium* was not able to convert DPA (n-3) to DHA. Surprisingly, we found the activities of Δ-6 and Δ-8 desaturases but the activities were relatively low, which indicated these two enzymes played little role in the fatty acid synthesis. As for three genes of PKS enzyme, they all possessed higher expression levels. As shown in Table 2 the pfaC which contains two DH (dehydrase) domains and one ER (enoyl-ACP reductase) showed higher sensitivity to the change of environment. The expression level decreased 57% and 92% during the transition from S1 to S2 and S3 to S4. As we known, DH catalyzes ketoacyl-ACP to enoyl-ACP and ER domain catalyzes the final step of fatty acid chain extension and reduces the double bond. The omission of the enoyl-ACP reduction determined the double bond formation of the unsaturated fatty acid. Cheng *et al*.^37^ proposed a method of screening high DHA yield *Aurantiochytrium* sp. strains with the inhibitors of enoyl-ACP reductase, and increased the DHA productivity and yield by 50% from 0.18 to 0.27 g/L h and 30% from 21 to 27 g/L, respectively, which also proved the importance of the final step of fatty acid chain extension. Therefore, the expression level of orfC might be helpful to explain the change the fatty acid composition during DHA fermentation.

As for the lipid turnover stage, to relieve the carbon starvation environment, cell accelerated the glucose regeneration process by degrading different kinds of polysaccharide. The expression of genes concerning EMP, PPP, TCA, fatty acid synthase and ATP generating pathways were all down-regulated as expected. While the glyceride degradation and conversion to polar lipid were relatively activate. Fatty acid degradation is the process by which energy is extracted from lipids. Similarly, in Tanaka’s study^38^ in oleaginous diatom *Fistulifera solaris*, the genes involved in β-oxidation were highly upregulated in the mitochondria, which induced cell to utilize massive amount of lipid for cell growth through TCA cycle, the gene of which were down-regulated but remains at relative high RPMK values. Besides, the porphyrin formation pathway was also found enhanced in the lipid turnover stage. As we known, porphyrin and its derivatives compounds were widely existed in the organelles and played significant roles in energy transfer^39^, which might also be related to the energy metabolism in lipid turnover stage. More importantly, we also found two transporters of PGR5 and SNQ2 which belongs to the ABC transporter in phosphatidylinositol signaling system were up-regulated. As an important intracellular second-messenger signaling system, phosohatidylinositol signaling system employs two second-messenger lipids, both of which are derived from phosphatidylinositol to change the extracellular signal to intracellular signal^40^. This active signal transduction process may also be closely related to the cell reaction to starvation condition and to the increase of polar lipid content in final fermentation results. Mahe *et al*.^41^ demonstrated that Pdr5 and Snq2 of *Saccharomyces cereviaiae* can mediate transport of steroid substrates, and Smart *et al*.^42^ found the expression of TUR2 gene, a Pdr5 homology, was induced by environmental stress treatments such as low temperature and high salt, which indicating that it may function during stress conditions. Therefore, we can speculate that expression of such two ABC transporters might be helpful in the environmental regulation.

## Conclusions

In this study, we used RNA-seq techniques to compare differential gene expression profiles at different fermentation stages, and got a series of potential gens related to the cell transition from cell growth to lipid accumulation and then to lipid turnover. Results showed that binding and single-organism process might be involved in the cell transition from cell growth to lipid accumulation while oxidation-reduction process played an important role in the transition from lipid accumulation to lipid turnover. As for the genes in the PKS pathway, pfaC showed higher sensitivity to the environmental change, which might be the key regulator for enhancing PUFA biosynthesis in the future. In addition, some other genes in signal transduction and cell transport were also revealed to be related to lipid turnover, which would enrich the current knowledge regarding lipid metabolism and help to enhance the DHA production and enrich different lipid fractions by *Schizochytrium*.

## Methods

### *Schizochytrium* sp. HX-308 collection and preparation

*Schizochytrium* sp. HX-308 (CCTCC M209059), an industrial strain stored in China Center for Type Culture Collection (CCTCC), was used for the present study. The seed culture medium and conditions were the same as those used in our previous study. The strain preserved at −80 °C was cultured for three generations and then transferred to a 5 L bioreactor which contains 3 L fermentation mediums. The fermentation medium was the same as our previous study. The impeller speed was set at 400 rpm at the beginning of fermentation and then reduced to 300 rpm when sodium glutamate was exhausted. The aeration was kept at 0.18 m^3^/h to achieve the aeration rate of 1 volume of air per volume of liquid per minute (vvm). The temperature was controlled at 30 °C. In the fermentation progress, glucose solution was fed into the bioreactor when the residual glucose concentration was below 20 g/L to keep the residual glucose concentration at 40 g/L. The fermentation time was conducted for 180 h.

### RNA isolation, library construction, and sequencing

Analysis of the developmental stage of *Schizochytrium* sp. HX-308 identified four developmental stages, cell growth stage (S1), early lipid accumulation stage (S2), later lipid accumulation stage (S3), and lipid turnover stage (S4) for transcriptome sampling. The samples of the four stages were collected at 18, 34, 72, 180 hours after culturing, respectively. The samples collected were immediately frozen in liquid nitrogen and stored at −80 °C until RNA extraction. The samples of the stages S1, S2, S3, S4 of *Schizochytrium* sp. HX-308 were used to construct four libraries.

Total RNA from *Schizochytrium* sp HX-308 was extracted using TRIzol reagent according to the manufacturer’s instructions (Invitrogen, USA). 0.75 mL of TRIzol ™ Reagent per 0.25 mL of sample was added to the pellet and the samples were homogenized by pipetting the lysate up and down. Then, the supernatant was collected after centrifugation (12,000 × g, 5 min, 4 °C) for 5 min. Afterwards, in order to precipitate and dissolved total RNA, isopropanol and nuclease-free water were added. Poly (A) mRNA was isolated from the collected total RNA using RNA beads with Oligo (dT). Mixed with the fragmentation buffer, the mRNA was fragmented into short fragments. Then cDNA was synthesized using the mRNA fragments as templates. Short fragments were purified and resolved with EB buffer for end reparation and single nucleotide A (adenine) addition. After that, the short fragments were connected with adapters. After agarose gel electrophoresis, the suitable fragments were selected for the PCR amplification as templates. Agilent 2100 Bioanaylzer and ABI StepOnePlus Real-Time PCR System were used in quantification and qualification of the sample library. The RNA libraries were sequenced on the sequencing platform of Illumina HiSeq^TM^ 2000.

### Analysis of sequencing results: mapping and differential expression

The raw reads were cleaned by removing reads with adapters, reads in which unknown bases are more than 10%, and low quality reads. Clean reads were mapped to a reference sequences with *SOAPaligner/SOAP2* allowing no more than 5 mismatches.

The gene expression level is calculated by using *RPKM* method for each sample.

The RPKM (reads per kb transcriptome per million mapped reads) is the most commonly used method to quantify gene expression. It can eliminate the influence of different gene length and sequencing discrepancy on the calculation of gene expression, which enable direct comparison of gene expression between samples.

Differential expression genes between two samples were identified using an algorithm developed according to reference. The false discovery rate (FDR) was used to determine the P-value threshold in multiple tests. An FDR ≤ 0.001 and the absolute value of Log_2_Ratio ≥1 were used to judge the significance of gene expression difference.

### Functional analysis of differentially expressed genes

Functional enrichment analysis, Gene Ontology (GO) and KEGG, were performed to identify DEGs which were significantly enriched in GO terms or metabolic pathways. GO terms of which corrected *p* value less than 0.05 were defined as significantly enriched GO terms in DEGs. For pathway enrichment analysis, pathways with Q value ≤ 0.05 are significantly enriched in DEGs.

### Fermentation performance analysis

Cell dry weight was determined by gravimetric method. 20 ml broth was transferred to a dried centrifuge tube by pipette and then centrifuged for 4 minutes at 4500 g. All the cells separated from the supernatant were transferred to the filter paper, and the filter paper was put into an oven at 60 °C to be dried. In the progress of drying, the weight was recorded repeatedly until the mass remained constant. 20 ml fresh fermentation broth was used to extract the lipid and then analyzed the individual fatty acid by GC-MS system (GC-2010, Shimadzu, Japan) according to our previous study^36^. Three replicates of each sample were analyzed to get the average value and standard deviations.

Glucose concentration was measured by a biosensor with glucose oxidase electrode (Institute of Biology, Shandong Academy of Sciences SBA-40C). The stirred bioreactor was equipped with sensors measuring temperature, dissolved oxygen (Mettler Toledo, Greifensee, Switzerland), and pH (Mettler Toledo, Greifensee, Switzerland).

### Quantitative real-time PCR (RT-qPCR) validation

The same samples with RNA-seq were also analyzed by RT-PCR. RNA was isolated using the Rapid fungal RNA extraction kit (Zoonbio Biotechnology, Nanjing, China). cDNA was obtained using the TUREscript cDNA Synthesis Kit (Zoonbio Biotechnology, Nanjing, China) and used for quantitative PCR analysis. Six target genes (FAS, orfA, orfB, orfC, acetyl-CoA carboxylase, malic enzyme) were tested in the present study. The relative levels of the amplified mRNAs were evaluated according to the 2^−△△Ct^ method using 18SrRNA for normalization^43–45^. Primers used in the experiments are listed in Table S5.

## Electronic supplementary material

supplementary info

supplementary dataset for the paper
