# Supplementary material for: Transcriptomic Analysis of the Regulation of Lipid Fraction Migration and Fatty Acid Biosynthesis in Schizochytrium sp
Source: Sci Rep. 2017 Jun 15;7:3562. doi: 10.1038/s41598-017-03382-9 (PMC5472558; doi:10.1038/s41598-017-03382-9)
Supplement: Supplementary file 1 — supplementary info [file 41598_2017_3382_MOESM1_ESM.doc]

**Transcriptomic Analysis of the Regulation of Lipid Fraction Migration and Fatty Acid Biosynthesis in *Schizochytrium* sp.**

Lujing Ren^1,2^, Xuechao Hu^2^, Xiaoyan Zhao^2^, ShenglanChen^2^, Yi Wu^4^, Dan Li^4^, Yadong Yu^1^, Lingjun Geng^2^, Xiaojun Ji^1,2^, He Huang^1,3^*

1, Jiangsu National Synergetic Innovation Center for Advanced Materials, 2, College of Biotechnology and Pharmaceutical Engineering, 3, School of Pharmaceutical Sciences, Nanjing Tech University, No. 30 South Puzhu Road, Nanjing 211816, People’s Republic of China

4, Xiamen Kingdomway Group company, No. 299 West Yangguang Road, Haicang, Xiamen 361022, China

*Corresponding author. Tel./fax: +86 25 58139942.

*E-mail*: [biotech@njtech.edu.cn](mailto:biotech@njtech.edu.cn) (H. Huang)

Table S1 Summary of draft reads of samples by Illumina sequencing

|  | S1 | S2 | S3 | S4 |
| --- | --- | --- | --- | --- |
| Clean reads | 23842182 | 23752928 | 23766084 | 23748300 |
| Total BasePairs | 2145796380 | 2137763520 | 2138947560 | 2137347000 |
| Genome map rate | 95.91% | 96.06% | 95.98% | 96.17% |
| Total mapped reads | 22868032 | 22817512 | 22811065 | 22837888 |
| Number of transcript | 8937 | 8911 | 8881 | 8988 |
| Number of novel transcript | 506 | 465 | 479 | 492 |
| Alternative Splicing | 1024 | 1008 | 1070 | 1253 |
| SNP | 1663 | 1728 | 1995 | 1919 |

Tab. S5 Primers for genes validated by Quantitative real-time PCR (qRT-PCR)

| Genes | Sequences |
| --- | --- |
| FAS-F | CCCAAGGGCAAGAAGACG |
| FAS-R | TGAGCCAGAAGCCGAGGT |
| PfaA-F | GAGCCCGCCGAAATCCT |
| PfaA-R | TGCCCTGCGAAGTGAAT |
| PfaB-F | GCAGGTTGTGCGTGAGTC |
| PfaB-R | GATACGGTTGCGGATGTT |
| PfaC-F | CACCGGCACTGTCAACCA |
| PfaC-R | GAGGCATGGAGTCGAAGG |
| ACC-F | GGCTGGCTCCTTTGGTA |
| ACC-R | GTTGATGCGGAAGTGGT |
| ME-F | AGACCCGCCACTCATACA |
| ME-R | CGCCGAGAAAGACAAAAG |
| AGPAT-F | GCCCAATGATGGACACCG |
| AGPAT-R | GTTTGAACTCCTTGAGAACACCC |
| TGL-F | CGTTCATAACAAGTTCACGCAGAT |
| TGL-R | GAGATAATGGTCCGATACCTCAAAA |
| 18S-F | ACGAGGTAGTGACGAGAAATA |
| 18S-R | ACAAAGATAGACGAGGATGG |

Figure legends:

Fig. S1. Overview of serial analysis of co-expressed genes identified by pairwise comparisons of the four transcriptomes. (a) Heatmap of co-expressed genes at four stages; (b) Venn diagram of the co-expressed genes at four stages. (c) Expression patterns of the genes in the eight main clusters, namely K1-K8, corresponding to the heatmap.

Fig. S2. Overview of DEGs by pairwise comparisons of the four transcriptomes, S1, S2, S3, S4. (a) Venn diagram of DEGs, (b) Number of up-regulated and down-regulated DEGs.

Fig. S3. GO terms significantly enriched in DEGs in comparisons of S1, S2, S3 and S4 libraries

Figure S4. Real-time quantitative RT-qPCR confirmation of 6 genes at the four stages, S1, S2, S3, S4. Relative gene expressions were normalized by comparison with the expression of 18sRNA, and analyzed using the 2 −ΔΔC T Method. The expression values were adjusted by setting the expression of S1 to be 1 for each gene.

Fig S1. Overview of serial analysis of co-expressed genes identified by pairwise comparisons of the four transcriptomes. (a) Heatmap of co-expressed genes at four stages; (b) Venn diagram of the co-expressed genes at four stages. (c) Expression patterns of the genes in the eight main clusters, namely K1-K8, corresponding to the heatmap.


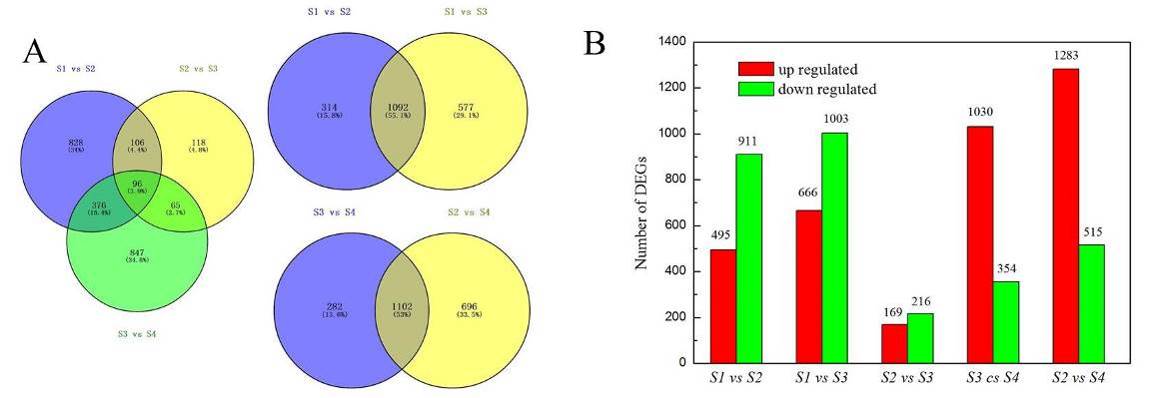


Fig. S2. Overview of DEGs by pairwise comparisons of the four transcriptomes, S1, S2, S3, S4. (a) Venn diagram of DEGs, (b) Number of up-regulated and down-regulated DEGs.


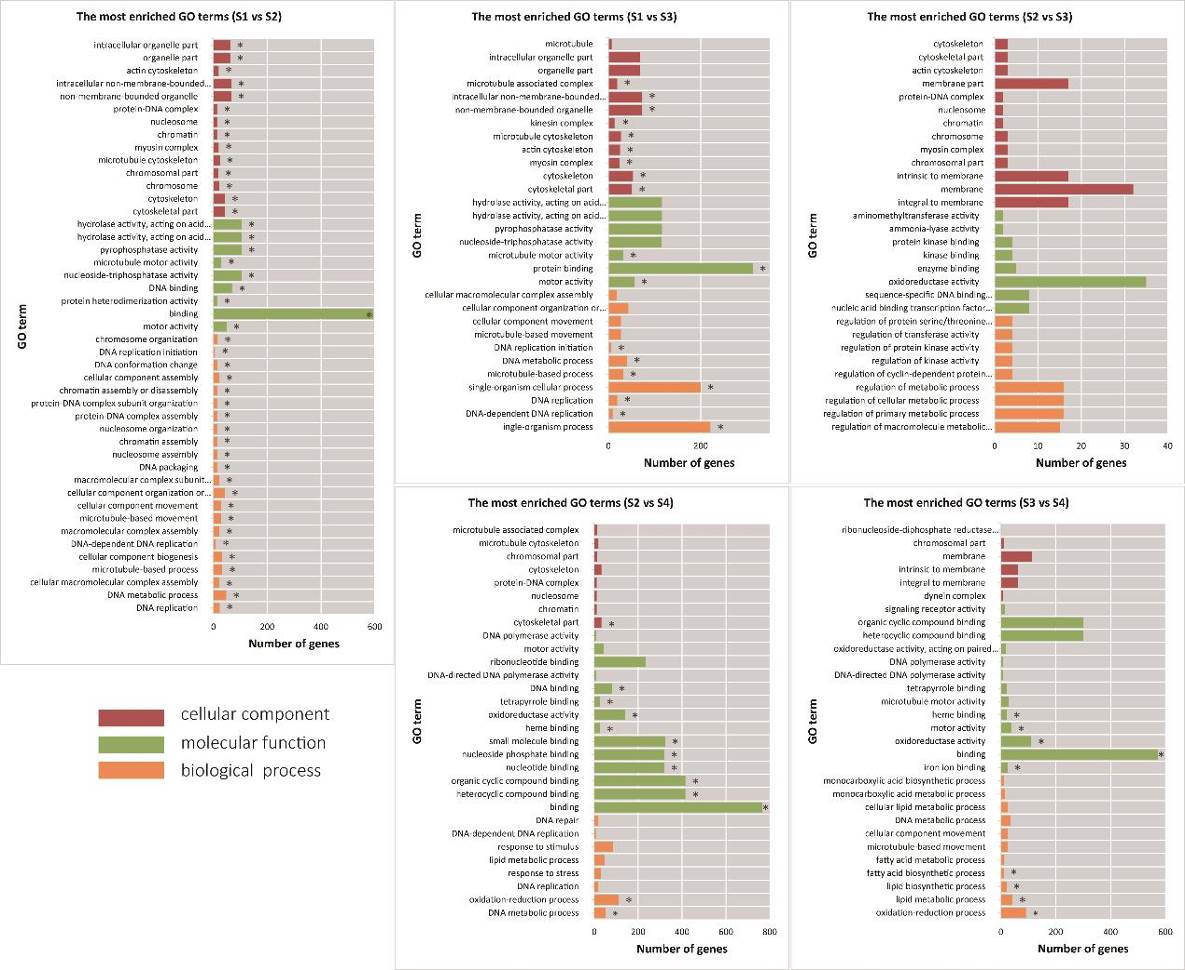


Fig. S3. GO terms significantly enriched in DEGs in comparisons of S1, S2, S3 and S4 libraries


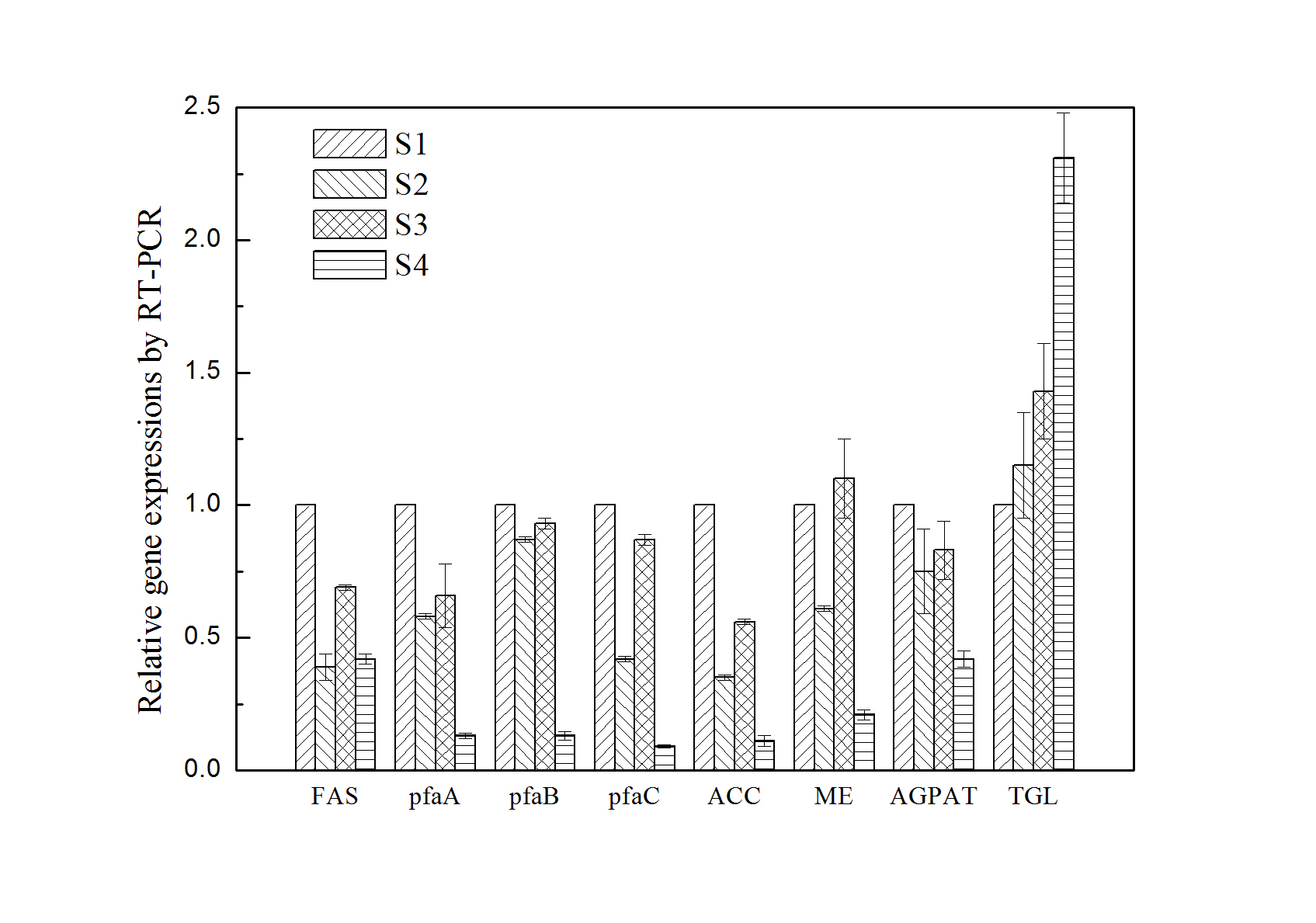


Figure S4. Real-time quantitative RT-qPCR confirmation of 6 genes at the four stages, S1, S2, S3, S4. Relative gene expressions were normalized by comparison with the expression of 18sRNA, and analyzed using the 2 −ΔΔC T Method. The expression values were adjusted by setting the expression of S1 to be 1 for each gene.
